# Characteristics of Endurance Competitions and Risk Factors for Elimination in New Zealand during Six Seasons of Competition (2010/11–2015/16)

**DOI:** 10.3390/ani9090611

**Published:** 2019-08-27

**Authors:** Kylie A. Legg, Jenny. F. Weston, Erica K. Gee, Charlotte F. Bolwell, Janis P. Bridges, Chris W. Rogers

**Affiliations:** 1 School of Veterinary Science, Massey University, Private Bag 11-222, 4442 Palmerston North, New Zealand; 2 School of Agriculture and Environment, Massey University, Private Bag 11-222, 4442 Palmerston North, New Zealand

**Keywords:** horse, endurance, competition, elimination, lameness

## Abstract

**Simple Summary:**

International media has recently raised awareness about horse welfare during endurance competitions. However, much of this attention is focused on international level competitions (FEI) and little is known about domestic level competitions and their risk factors for elimination. The characteristics of endurance rides and risk factors for elimination of horses due to lameness and metabolic reasons were described by assessing the records of all competitors during six competition seasons in New Zealand (2010/11–2015/16). Endurance ride entries were dominated by lower distances (40–80 km), with the number of eliminations increasing with ride distance. The competition season was structured with the longer, more competitive rides at the end of the season, allowing the shorter, earlier rides to be used as conditioning rides. Ride distance, location and progression of the endurance season were significantly associated with eliminations due to lameness or metabolic reasons and horse age was significant for metabolic reasons only. The changing profile of endurance competitors over the years showed a decreasing number of higher level riders and subsequent increase in lower level riders competing in shorter rides. This profile with low competition speeds, demonstrates better horse welfare outcomes than seen in other parts of the world.

**Abstract:**

The welfare of horses in endurance competitions has been the focus of recent media attention. Epidemiological studies have examined the sport at the international (FEI) level. However, much of the participation in the sport occurs at a national level in preparation for FEI level competition. The aims of this study were to describe participation in, and risk factors for elimination, from New Zealand endurance competitions. Data were collated from all endurance competitions (≥40 km) held in New Zealand during the 2010/11–2015/16 competition seasons. There were 6885 starts (n = 775 horses, n = 665 riders), horses had a median age of 9 years (IQR 6.2–10.0) and had a median of 3 (IQR 2–5) starts per season. Accumulated ride distance per season per horse decreased from a median of 240 km/horse (IQR 120–440) in 2010/11 to 180 km/horse (IQR 80–320) in 2015/16. Ride entries were dominated by the 40 km (n = 2834, 41%) and 80 km (n = 2517, 37%) distances. Eliminations increased with ride distance, from 7% in 40 km rides to 53% in the 160 km rides. Lameness accounted for the majority of eliminations (64%). The odds of elimination due to lameness were significantly associated with ride distance, location (North or South island) and time of year. The 11% of starters eliminated for metabolic reasons of the horse had increased odds of elimination associated with horse age, ride distance, location and time of year.

## 1. Introduction

Endurance rides are long distance competitions ridden over natural terrain, with distances ranging from 40 km to 160 km. The horses are subjected to regular veterinary inspections at least every 40 km to ensure their fitness to start and to continue. Distances longer than 40 km are completed as multi-loop rides, with the expectation that an 80 km competition will be split into three loops and a 160 km competition will be run over 5–6 loops. Therefore there is an increasing frequency of veterinary inspection over the longer distances. In New Zealand, a qualification system is imposed on all horses and riders who are new to these competitions, even when the young or new horse is being ridden by an experienced rider [1]. This system limits the speed at which the horses can compete in the initial stages of their career (introductory or novice classes) to protect their welfare. The horse can then progress to intermediate and open level competitions. If a horse has been out of competition for more than two years, they drop to a lower level of competition and must prove themselves at that level before progressing.

The growing popularity of endurance riding worldwide has resulted in increased attention in the media regarding the welfare of endurance horses [2,3]. In particular, two different types of endurance riding seem to be evolving around the world; a more traditional endurance ride over challenging natural terrain where horses are commonly competing between 10 and 18 kmh^−1^ and less technical courses where most horses race at >20 kmh^−1^ [4,5,6]. Epidemiological studies have examined the sport at Fédération Equestre Internationale (FEI) level events to identify risk factors contributing to elimination [7,8,9,10]. However, much of the participation in the sport occurs at a national level in preparation for FEI level sport [11,12], which may not reflect the same competition structure and risk factors characteristic of international level competition.

Internationally, elimination rates in endurance competitions range from 20% to 60% [8,9,10,13,14]. Lameness accounts for the majority of eliminations (50–70%) with metabolic problems accounting for 15–25% of all eliminations [10,13,14]. Risk factors for elimination due to lameness or metabolic reasons are multifactorial and include: country, venue, number of entries in the competition, speed, ride length, track conditions, breed, age and horses’ prior experience [6,7,13,14,15]. Other reasons for elimination include surface factors (injuries of the horse from the saddle, bridle or superficial leg injuries), course error, rider injury, rule violation and withdrawal from competition.

National (governed by Equestrian Sports New Zealand) and international level (FEI) endurance in New Zealand have been closely aligned for over 20 years [12]. The FEI have implemented new regulations to improve horse welfare based on data, with a focus on mandatory rest periods between successive competitions [16]. Further regulations are proposed to limit speed, at least in the early stages of the horses’ international level competition. Recent studies of the characteristics of the endurance population in New Zealand have indicated that most participants are amateurs (i.e., they do not derive their main income from the sport) with a focus on participation, and who own and train their horses themselves [11,12]. The competition season in New Zealand begins in the spring (August/September) and ends in autumn (April/May), with the majority of riders in New Zealand using competitions as training opportunities for their horses [12]. It is possible that the risk factors for elimination may differ at national level competition, particularly when horses are competing at lower speeds. These competition characteristics suggest that international regulations pertaining to crewing, trainers and speed restriction [16] may not be necessary to protect the welfare of horses in the slower, more traditional endurance competitions that occur in New Zealand and some other countries.

The aim of this paper was to describe the participation in endurance competitions (domestic and FEI level) during six complete seasons in New Zealand. A secondary aim was to assess risk factors for elimination of the horses during these rides due to lameness or metabolic reasons.

## 2. Materials and Methods

All horses competing in official endurance rides within New Zealand have their entry and ride details logged with the official national registration body, Equestrian Sports New Zealand (ESNZ). These data are maintained as either paper copies (and loaded online as a PDF) or as a dynamic html table. Data were collated within a customised Access database from electronic and paper records of all Endurance competitions (minimum distance 40 km) held within New Zealand during the 2010/11–2015/16 competition seasons. Ride details were cross referenced within the online ESNZ database to extract the demographic details for each horse (age, sex and previous cumulative kilometres in official rides). Horses that competed on a casual basis (single day registration) were excluded from further analysis in this study.

Ride distance was recorded according to the closest official ride category (40–49 km = 40 km, 50–65 km = 60 km, 78–88 km = 80 km, 90–105 km = 100 km, 120–124 km = 120 and 160 km). Most ride distances were a multiple of 10 km, although some events differed due to the difficulty in securing loops of specific distances.

Descriptive analysis was carried out for the variables of number of starts, completions, eliminations and ride speeds, and included calculation of the mean, median, IQR and minimal and maximal values. Normality was assessed using an Anderson-Darling test (for >5000 observations) with Pearson’s Chi-squared test for differences between groups.

The exposure variables associated with ride elimination due to lameness reasons and due to metabolic reasons included horse-level variables; horse age and sex, as well as race-level variables; ride distance (<100 km or ≥100 km), competition season, competition level (national or international), rider age (junior or senior), rider gender, location (North or South Island of New Zealand) and month of year. Screening of all exposure variables for elimination due to lameness and elimination due to metabolic reasons was performed separately using univariable logistic regression. Variables with *p* < 0.2 were considered for inclusion in the multivariate models. Exposure variables were then assessed for collinearity and those with the strongest association with the outcome variable were used in the final multivariate models. The multivariate models were built using a forward selection procedure whereby variables with a Wald-test *p* ≤ 0.05 were retained in the model.

All statistical analyses were conducted using RStudio (version 3.5.1, 2018; R Foundation for Statistical Computing, Vienna, Austria), with the level of significance set at *p* < 0.05.

## 3. Results

### 3.1. Participants

During the 2010/11–2015/16 competition seasons there were a total of 7491 starts in endurance rides of ≥40 km in New Zealand. Of the 92% (n = 6885) starts that had full registration details with ESNZ, the governing body for endurance in New Zealand, there were 775 horses and 665 riders. The remainder, 8% (n = 606) of starts, were from 91 horses that competed on a casual day (single competition start) permit which restricted them to rides of 46 km or less [1] and were excluded from subsequent analysis. Of the horses registered with ESNZ, there were 425 (55%) geldings, 338 (44%) mares, 11 (1%) stallions and 1 unrecorded, with a median age of 9 (IQR = 6–10) years.

### 3.2. Starts

Ride entries were dominated by the 40 km (n = 2834, 41%) and 80 km (n = 2517, 37%) distances, with 16% (n = 1085) of all starts in competitions of 100 km or greater (Table 1). There were fewer longer distance (≥100 km) races completed at the beginning of the endurance competition season than at the end of the season (5% vs. 33%), reflecting the structure of the competition season. Median completion speed for all distances was higher for open horses than lower level horses with higher speeds observed for the longest distance rides (160 km) of 14.6 kmh^−1^ (IQR 13.2–15.9 kmh^−1^). Across all seasons, the percentage of horses which were eliminated due to lameness or metabolic reasons increased with distance (Table 1).

There were 46–68 competition events each season, with the 2012/13 season having the highest number of competitions and 2014/15 the lowest. There was a moderate decrease in the annual numbers of starts per season from 1271 in 2010/11 to 961 in 2015/2016. However, the number of open level competitors more than halved over the six seasons, with a concurrent increase in the number of intermediate and novice competitors (Table 2). This was reflected in the number of starters in the longer distance rides (>100 km) decreasing substantially over the six seasons (Table 2). There was a decrease in the number of horses that had a start from 327 in 2010/11 to 286 in 2015/2016. The overall median number of starts per horse per season remained relatively constant at 3 (IQR = 2–5), with a significant drop in starts per horse to a median of 2 (IQR = 1–4, *p* < 0.001) in the 2014/5 season. 38% (n = 297/775) of horses had starts in three or more seasons, with 44 horses (6%) starting in all six seasons.

The median accumulated ride distance per season per horse decreased from 240 km/horse (IQR = 120–440) in 2010/11 to 180 km/horse (IQR = 80–320) in 2015/16 (*p* < 0.001). Horses had a median of 22–28 days (IQR 8–71 and 14–50, respectively) between competitions following a 40 km race unless the next distance was 160 km where the median was 14 days (IQR 13–69). There was a median of 34–93 days following races ≥ 100 km, generally with an increasing break following a larger first distance. There was a median of 63 days (IQR 55–90) between two consecutive 160 km rides.

### 3.3. Risk Factors for Elimination

During the 2010/11–2015/16 endurance seasons, 16% (n = 1038) of horses failed to complete a ride. The percentage of horses that failed to complete a ride ≥ 100 km was 37% (n = 401/1085). There were 778 eliminations due to lameness or metabolic reasons, comprising 75% of all non-completions during the 2010/11–2015/16 endurance seasons. 64% (n = 664) of all eliminations were due to lameness and 11% (n = 110) were due to metabolic reasons. Univariable screening of variables identified five variables for inclusion (*p* < 0.2) in a multivariable model predicting elimination due to lameness (Table 3). These included horse age, ride distance (<100 km or ≥100 km), competition level (national or international FEI), location (North or South island of New Zealand) and month of year. Competition level had significant collinearity with ride distance (χ^2^ = 3844, *p* < 0.001) so was omitted from further analysis as ride distance had a stronger association with the outcome variable.

Univariable screening of variables identified six variables for inclusion (*p* < 0.2) in a multivariable model predicting elimination due to metabolic reasons (Table 4). These included horse age, ride distance (<100 km or ≥100 km), competition season, competition level (national or international FEI), location (North or South island of New Zealand) and month of year.

### 3.4. Multivariable Logistic Regression

The multivariable model identified increased odds of elimination due to lameness with longer distances, competition location in the South Island and progression of the competition season (Table 5). Competitions at the beginning of the competition season (August) had lower odds of elimination.

The multivariable model identified increased odds of elimination due to metabolic reasons with horse age, longer distances, the North Island, November, and the end of the competition season (Table 6).

## 4. Discussion

This was the first study to describe the participation and competition structure of endurance rides in New Zealand in addition to risk factors for elimination due to lameness or metabolic reasons. The majority of participants took part in 40 or 80 km rides with 2–3 starts per season. This profile represented the large proportion of amateur riders competing in the sport and the use of these lower distance rides as conditioning rides for FEI level events later in the season [12]. This is reflected by the greater number of longer (≥100 km) distance rides towards the end of the competition season (March–July) as compared to the beginning, reflecting the classic structure of the competition season in New Zealand with the pinnacle FEI rides and championship events towards the end of the season.

There was a decreasing number of open and thus longer distance starts over the six seasons, although the number of lower level competitors increased. This was supported by the decrease in median accumulated ride distance per season per horse across the six study seasons from 240 km/horse–180 km/horse. The median number of starts per horse remained relatively constant across the seasons (with the exception of 2014/15), indicating that ride distances per horse decreased across the seasons. During the 2014/15 season, there were fewer competitions available, possibly accounting for the smaller number of starts per horse that season. The reasons for the decline in competitors in open and longer distance events are not clear. The introduction of mandatory rest periods in FEI endurance rules and these being incorporated into the ESNZ endurance rules [1,16] may cause participants not to compete close to a championship event where elimination would cause them to become ineligible to compete at a pinnacle event. Sales of horses overseas and retirement of seasoned horses could be a contributing factor, as experienced riders developing a younger horse are ineligible to compete in the longer distances for several years. Increases in costs, both directly and indirectly associated with the sport (e.g., fuel and transport costs) may also impact riders’ discretionary spending.

Completion speeds were at the lower end of international standards (14–30 kmh^−1^ for international 160 km rides) [7,8]. These speeds could be affected by the use of rides as training opportunities [12] and the use of variable and hilly terrain for rides inherently providing a restriction on speed. Additionally, many events are held over farmland which may include a requirement to open and close gates whilst on course, further slowing speeds. Completion speeds increased with distance in the open category remaining constant across the middle distances. This indicated that the more experienced horses progressed from shorter conditioning rides early on in the season, to longer, faster and more competitive rides at the end of the season. The fact that championship events are held towards the end of the season further confounds these results as riders are more likely to try to be competitive when there is a national title at stake. The large number of novice riders in the lower distance rides maintained fairly constant speeds across the distances, remaining within their speed limitation of 13.5 kmh^−1^ [1].

The temporal relationship between two consecutive starts indicated that riders entered into shorter distance competitions with a month or less until a second competitive ride. The length of time following longer distance rides was greater (1–3 months), indicating that more time off was allowed to horses having competed these rides (or that this was their pinnacle event). This aligned with the prescribed mandatory rest periods assigned to the different distances, with 5 and 12 days for 40 and 80 km rides respectively and increasing incrementally up to 33 days for rides > 146 km [1]. The risk of elimination due to lameness has been shown to reduce with a periods of more than 90 days since a horses’ previous FEI ride and the introduction of mandatory rest periods in the FEI rules have demonstrated clear benefits [15,17]. Data indicated that rest periods in New Zealand were more conservative than the guidelines ruled, but could be affected by rider location, competition schedule and adverse weather cancellations restricting competition opportunities.

### Elimination

Elimination rates increased with increasing ride distance and speed in line with data published examining FEI rides [18]. The percentage of eliminated horses in the present study for rides ≥ 100 km (37%) was less than that published in international studies of ~50% [7,19]. Unsurprisingly, the shorter length rides have a lower number of eliminations (total average elimination for all distances = 16%), which was similar to elimination rates from national level rides in the USA [8,13]. This could be due to the aims of competitors in lower level competitions being for pleasure or training rather than to qualify or get the best possible placing. The percentage of horses eliminated for lameness or metabolic reasons was not significantly different across the six study seasons. This indicates that there has been little change in the competition or training practices across the study period. Therefore, this indicates that horse welfare has not been negatively compromised by inadequate guidelines as is suggested on an international scale [2]. In contrast to the increasing speed of top level competitions and subsequent increase in orthopaedic injuries reported internationally [4], competition speeds and elimination rates in New Zealand remained relatively constant across the six seasons.

A greater ride distance was likely to have a significant effect on eliminations for both lameness and metabolic reasons. This was in agreement with international studies [7,13]. Logically, a greater amount of exercise places an increased load on the musculoskeletal system (and respiratory system), and, coupled with muscular fatigue, would lead to increased risk of elimination due to lameness. In addition, longer rides were likely to be more competitive than the shorter distance rides used for preparation, possibly resulting in riders pushing their horses harder than they might during preparatory rides.

Location was associated with the risk of elimination due to both lameness and metabolic reasons. Horses competing in the South Island of New Zealand had a higher risk of elimination due to lameness than those in the North Island, which had a higher risk of elimination due to metabolic reasons. This may be attributable to a number of factors including terrain (South Island has rougher terrain), climate (warmer in the North Island) or training methods between the two islands, all of which are avenues for further investigation.

Time of year had a significant effect on the risk of elimination due to both lameness and metabolic reasons with the beginning of the season (August–October) having the lowest risk for both reasons. Risk of elimination due to lameness increased as the season progressed until April/May. This was likely an effect of the progressive loading of training and competitions throughout the season in addition to the higher number of horses starting in longer distance competitions later in the season. Furthermore, the summer months (November to March) coincide with warmer, dryer weather, resulting in hard ground, likely to increase the concussive forces on the horses’ locomotor system. There was an increased risk of elimination due to metabolic reasons in November and March–May. This was likely due to the longer distance rides offered at these times of year, but could also reflect the advent of summer in November, and the beginning of cooler weather in March–May. The changing temperatures and increase of dust/pollen in the environment at these times of year may adversely affect the horses’ respiratory systems. Additionally, the championship events (North Island, South Island and National Championships) include the majority of longer distance rides and are held between January and Easter. Riders are likely to ride more competitively and thus faster, at these events, and the higher elimination rates from these longer distance rides are more in line with those found in the international literature [10,14]. Longer distance rides also include a proportion of the event that is ridden in the dark, most commonly in the earlier stages of the ride, making it more difficult to judge the terrain and thereby increasing the risk of a horse becoming lame.

Risk of elimination due to metabolic reasons increased with increasing horse age, similar to previous studies [9,13]. This may be related to the minimum age limits set for competitions in New Zealand (minimum 6 years old for rides ≥ 100 km and 7 years for rides ≥ 140 km) and a maximum speed restriction for 4 year old horses of 12 kmh^−1^ [1]. For this reason, 8 years of age was chosen as the lower category range in the multivariate analysis. These restrictions may encourage more conservative racing strategies in younger horses and thus a lower risk of elimination for these horses.

## 5. Conclusions

Endurance competitions in New Zealand are attended by a diverse population of horses and riders, the majority of which participate in shorter distance rides, with slow speeds and few starts during the season. This reflects the amateur profile of New Zealand competitors and their use of shorter distance rides as conditioning rides for the more competitive, longer distance rides later in the season. The number of open level (and longer distance) competitors decreased over the study period, whilst the number of lower level competitors increased, reflecting the changing profile of the sport in New Zealand. Both speed and elimination rate increased with ride distance. Ride distance, location and month of year significantly affected risk of elimination due to lameness or metabolic reasons, whilst horse age was a significant factor for risk of elimination due to metabolic reasons only. This profile provides a basis for the adaptation of international regulations specific to endurance rides in New Zealand and confirms that endurance rides ridden at slower speeds over technically challenging terrain have fewer eliminations and better horse welfare.

## Figures and Tables

**Table 1:** Characteristics of competition distances over six endurance competition seasons 2010/11–2015/6 in New Zealand.

		Competition Distance (km)						*p*
		40	60	80	100	120	160	Value
Horses	**n**	729	236	529	226	199	103	<0.001
Starts	**n**	2834	449	2517	399	452	234	<0.001
**Completions:**								
Beginning (Aug–Nov)	**n** **(%)**	1707 (55%)	154 (5%)	1100 (35%)	75 (2%)	95 (3%)	0 (0%)	<0.001
Middle (Dec–Feb)	**n** **(%)**	792 (32%)	200 (8%)	1016 (40%)	215 (9%)	210 (8%)	80 (3%)	<0.001
End (Mar–Jul)	**n** **(%)**	335 (27%)	95 (8%)	401 (32%)	109 (9%)	147 (12%)	154 (12%)	<0.001
Total	**n** **(%)**	2636 (93%)	378 (84%)	2101 (84%)	283 (71%)	292 (65%)	109 (47%)	<0.001
**Speed (kmhr^−1^):**								
Novice/Int *	**Median** **IQR**	11.0 9.7–12.3	11.5 10.3–12.8	11.3 10.3–12.3	10.7 10.0–11.3	n/a	n/a	<0.001
Open *	**Median** **IQR**	12.7 11.3–14.4	13.7 12.3–15.3	13.2 11.7–14.8	13.6 12.3–15.3	13.7 12.6–15.1	14.6 13.2–15.9	<0.001
**Elimination lameness**	**n** **(%)**	92 (3%)	40 (9%)	267 (11%)	88 (22%)	100 (22%)	82 (35%)	<0.001
**Elimination metabolic**	**n** **(%)**	21 (1%)	10 (2%)	35 (1%)	6 (2%)	25 (6%)	22 (9%)	<0.001

* Novice classes are speed limited, whereas intermediate/open classes are not. Intermediate classes are ≤80 km.

**Table 2:** Number of starters per season over six endurance competition seasons 2010/11–2015/6 in New Zealand.

	Season					
Number of Starters	2010/2011	2011/2012	2012/2013	2013/2014	2014/2015	2015/2016
Distance (km):						
40	412	530	568	461	428	435
60	71	67	82	113	41	75
80	465	477	600	412	234	329
100	138	75	89	39	30	28
120	132	84	64	61	52	59
160	53	29	50	29	38	35
Total	1271	1262	1453	1115	823	961
Ride class:						
Novice/Int *	390	432	559	475	533	608
Open	881	830	894	638	290	353
Horses (n)	327	322	347	315	306	286

* Novice/Intermediate competitors.

**Table 3:** Univariate logistic regression of associations between ride elimination (No/Yes) due to lameness reasons only and the predictor variables; horse age and sex, ride distance (<100 km or ≥100 km), competition season, competition level (national or international), rider age (junior or senior), rider gender, location (North or South island of New Zealand) and month of year.

Variable	Category	Eliminated No	Eliminated Yes	OR	95% CI	*p*	*p* (Wald)
		n (%)	n (%)				
Horse age	≤8	1871 (92%)	155 (8%)	Ref			<0.001
	8–10	1876 (90%)	216 (10%)	1.39	1.12–1.73	0.003	
	>10	2465 (89%)	298 (11%)	1.46	1.19–1.79	<0.001	
Horse sex	F	2771 (90%)	304 (10%)	Ref			0.70
	M	3444 (90%)	465 (10%)	0.97	0.82–1.13	0.67	
Ride distance	<100 km	5401 (93%)	399 (7%)	Ref			<0.001
	≥100 km	815 (75%)	270 (25%)	4.48	3.78–5.32	<0.001	
Competition season	2010/2011	1152 (91%)	119 (9%)	Ref			0.43
	2011/2012	1149 (91%)	113 (9%)	0.91	0.71–1.17	0.72	
	2012/2013	1305 (90%)	148 (10%)	1.04	0.82–1.32	0.47	
	2013/2014	997 (89%)	118 (11%)	1.03	0.80–1.33	0.32	
	2014/2015	754 (92%)	69 (8%)	1.04	0.78–1.37	0.44	
	2015/2016	859 (89%)	102 (11%)	1.12	0.87–1.46	0.33	
Competition level	National	5203 (93%)	372 (7%)	Ref			<0.001
	International	1013 (77%)	297 (23%)	4.10	3.47–4.84	<0.001	
Rider age	Junior	790 (91%)	81 (9%)	Ref			0.87
	Senior	5309 (90%)	560 (10%)	1.03	0.81–1.32	0.82	
Rider gender	F	4758 (90%)	502 (10%)	Ref			0.41
	M	1458 (90%)	167 (10%)	1.09	0.90–1.30	0.38	
Location (Island)	North	4299 (91%)	422 (9%)	Ref			0.001
	South	1917 (89%)	247 (11%)	1.31	1.11–1.55	0.001	
Month	August	276 (98%)	7 (2%)	0.41	0.17–0.83	0.02	<0.001
	September	688 (96%)	29 (4%)	0.67	0.43–1.04	0.08	
	October	1103 (94%)	69 (6%)	Ref			
	November	879 (92%)	80 (8%)	1.45	1.04–2.04	0.03	
	December	880 (89%)	104 (11%)	1.89	1.38–2.60	<0.001	
	January	740 (86%)	116 (14%)	2.51	1.84–3.44	<0.001	
	February	601 (89%)	72 (11%)	1.92	1.36–2.71	<0.001	
	March	591 (86%)	98 (14%)	2.65	1.92–3.68	<0.001	
	April	291 (78%)	83 (22%)	4.56	3.23–6.45	<0.001	
	May	94 (91%)	9 (9%)	1.53	0.69–3.01	0.25	
	June	42 (95%)	2 (5%)	0.76	0.12–2.54	0.71	
	July	31 (100%)	0 (0%)	0.00	0.00–1.02	0.96	

**Table 4:** Univariate logistic regression of associations between ride elimination (No/Yes) due to metabolic reasons only and the predictor variables; horse age and sex, ride distance (<100 km or ≥100 km), competition season, competition level (national or international), rider age (junior or senior), rider gender, location (North or South island of New Zealand) and month of year.

Variable	Category	Eliminated No	Eliminated Yes	OR	95% CI	*p*	*p* (Wald)
		n (%)	n (%)				
Horse age	≤8	2007 (99%)	19 (1%)	Ref			<0.001
	8–10	2058 (98%)	34 (2%)	1.75	1.00–3.13	0.053	
	>10	2697 (98%)	66 (2%)	2.58	1.60–4.44	0.000	
Horse sex	F	3024 (98%)	51 (2%)	Ref			0.758
	M	3741 (98%)	68 (2%)	1.08	0.75–1.56	0.688	
Ride distance	<100 km	5734 (99%)	66 (1%)	Ref			<0.001
	≥100 km	1032 (95%)	53 (5%)	4.46	3.08–6.43	<0.001	
Competition season	2010/2011	1245 (98%)	26 (2%)	Ref			0.021
	2011/2012	1244 (99%)	18 (1%)	0.69	0.37–1.26	0.235	
	2012/2013	1434 (99%)	19 (1%)	0.63	0.34–1.15	0.135	
	2013/2014	1103 (99%)	12 (1%)	0.52	0.25–1.02	0.064	
	2014/2015	799 (97%)	24 (3%)	1.44	0.82–2.53	0.205	
	2015/2016	941 (98%)	20 (2%)	1.02	0.56–1.83	0.953	
Competition level	National	5514 (99%)	61 (1%)	Ref			<0.001
	International	1252 (96%)	58 (4%)	4.19	2.90–6.03	<0.001	
Rider age	Junior	859 (99%)	12 (1%)	Ref			0.530
	Senior	5767 (98%)	102 (2%)	1.27	0.72–2.44	0.443	
Rider gender	F	5171 (98%)	89 (2%)	Ref			0.758
	M	1595 (98%)	30 (2%)	1.09	0.71–1.64	0.677	
Location (Island)	North	4628 (98%)	93 (2%)	Ref			0.030
	South	2138 (99%)	26 (1%)	0.61	0.38–0.92	0.025	
Month	August	283 (100%)	0 (0%)	0.00	0–0.38	0.972	<0.001
	September	714 (100%)	3 (0%)	0.61	0.13–2.12	0.468	
	October	1164 (99%)	8 (1%)	Ref			
	November	941 (98%)	18 (2%)	2.78	1.24–6.81	0.017	
	December	975 (99%)	9 (1%)	1.34	0.51–3.59	0.545	
	January	839 (98%)	17 (2%)	2.95	1.30–7.26	0.012	
	February	665 (99%)	8 (1%)	1.75	0.64–4.78	0.265	
	March	664 (96%)	25 (4%)	5.48	2.57–13.05	0.000	
	April	348 (93%)	26 (7%)	10.87	5.10–25.89	0.000	
	May	98 (95%)	5 (5%)	7.42	2.21–22.68	0.001	
	June	44 (100%)	0 (0%)	0.00	0–inf	0.989	
	July	31 (100%)	0 (0%)	0.00	0–inf	0.991	

**Table 5:** Multivariate logistic regression model of associations between ride elimination (No/Yes) due to lameness only and predictor variables; ride distance, location and time of year.

Variable	Category	Estimate	SE	OR	95% CI	*p*	*p* (Wald)
Intercept		−2.84	0.13	0.06	0.04–0.07	<0.001	
Ride distance	<100 km	Ref					<0.001
	≥100 km	1.23	0.10	3.42	2.81–4.14	<0.001	
Location (island)	North	Ref					0.007
	South	0.24	0.09	1.27	1.07–1.52	0.007	
Month	August	−0.87	0.40	0.42	0.17–0.86	0.03	<0.001
	September	−0.42	0.23	0.66	0.42–1.01	0.06	
	October	Ref					
	November	0.03	0.18	1.03	0.73–1.46	0.85	
	December	0.43	0.16	1.54	1.12–2.13	0.008	
	January	0.43	0.17	1.54	1.11–2.14	0.01	
	February	0.22	0.18	1.25	0.87–1.79	0.23	
	March	0.55	0.17	1.74	1.24–2.44	0.001	
	April	0.72	0.19	2.05	1.24–3.00	<0.001	
	May	0.09	0.38	1.10	0.49–2.20	0.80	
	June	−0.20	0.73	0.82	0.13–2.74	0.79	
	July	−12.84	261.09	0.00	0.00–0.96	0.96	

**Table 6:** Multivariate logistic regression model of associations between ride elimination (No/Yes) due to metabolic reasons only and predictor variables; horse age, ride distance, location and time of year.

Variable	Category	Estimate	SE	OR	95% CI	*p*	*p* (Wald)
Intercept		−5.24	0.41	0.01	0.00–0.01	<0.001	
Horse age	≤8	Ref					<0.001
	8–10	0.29	0.29	1.34	0.76–2.43	0.32	
	>10	0.67	0.27	1.96	1.18–3.39	0.01	
Ride distance	<100 km	Ref					<0.001
	≥100 km	0.88	0.21	2.41	1.59–3.65	<0.001	
Location (island)	North	Ref					0.03
	South	−0.48	0.23	0.62	0.39–0.96	0.04	
Month	August	−13.57	385.16	0.00	0.00–0.35	0.98	<0.001
	September	−0.44	0.68	0.65	0.14–2.25	0.52	
	October	Ref					
	November	0.81	0.43	2.24	0.99–5.54	0.06	
	December	0.15	0.49	1.16	0.44–3.13	0.76	
	January	0.63	0.45	1.88	0.80–4.75	0.16	
	February	0.38	0.51	1.46	0.53–4.04	0.46	
	March	1.37	0.42	3.92	1.79–9.51	0.001	
	April	1.74	0.44	5.70	2.51–14.23	<0.001	
	May	1.59	0.59	4.89	1.42–15.29	0.007	
	June	−13.70	977.56	0.00	0.00–inf	0.99	
	July	−13.38	1164.31	0.00	0.00–inf	0.99	
